# Regulatory T Cells in Arterivirus and Coronavirus Infections: Do They Protect Against Disease or Enhance it?

**DOI:** 10.3390/v4050833

**Published:** 2012-05-15

**Authors:** Thomas E. Cecere, S. Michelle Todd, Tanya LeRoith

**Affiliations:** Department of Biomedical Sciences and Pathobiology, Virginia-Maryland Regional College of Veterinary Medicine, Virginia Tech, Blacksburg, VA 24061, USA; Email: tcecere@vt.edu (T.C.); smtodd@vt.edu (S.T.)

**Keywords:** regulatory T cell, arterivirus, porcine reproductive and respiratory syndrome virus, lactate dehydrogenase-elevating virus, coronavirus

## Abstract

Regulatory T cells (T_regs_) are a subset of T cells that are responsible for maintaining peripheral immune tolerance and homeostasis. The hallmark of T_regs_ is the expression of the forkhead box P3 (FoxP3) transcription factor. Natural regulatory T cells (nT_regs_) are a distinct population of T cells that express CD4 and FoxP3. nTregs develop in the thymus and function in maintaining peripheral immune tolerance. Other CD4^+^, CD4^-^CD8^-^, and CD8^+^CD28^-^ T cells can be induced to acquire regulatory function by antigenic stimulation, depending on the cytokine milieu. Inducible (or adaptive) T_regs_ frequently express high levels of the interleukin 2 receptor (CD25). Atypical T_regs_ express FoxP3 and CD4 but have no surface expression of CD25. Type 1 regulatory T cells (Tr1 cells) produce IL-10, while T helper 3 cells (Th3) produce TGF-β. The function of inducible T_regs_ is presumably to maintain immune homeostasis, especially in the context of chronic inflammation or infection. Induction of T_regs_ in coronaviral infections protects against the more severe forms of the disease attributable to the host response. However, arteriviruses have exploited these T cell subsets as a means to dampen the immune response allowing for viral persistence. T_reg_ induction or activation in the pathogenesis of disease has been described in both porcine reproductive and respiratory syndrome virus, lactate dehydrogenase elevating virus, and mouse hepatitis virus. This review discusses the development and biology of regulatory T cells in the context of arteriviral and coronaviral infection.

## 1. Introduction

Regulatory T cells (T_regs_) provide a balance between combating pathogens and the risk of developing autoimmunity or overwhelming inflammation. Regulatory T cells can be divided into two groups—natural T_regs_ develop in the thymus, while inducible T_regs_ are generated in the periphery from conventional T cells in response to different stimuli. The natural T_regs_ are the best characterized of the two groups and make up approximately 5–10% of circulating T lymphocytes in mice and humans [1]. Regulatory T cells are primarily characterized by the expression of the transcription factor FoxP3, although conventional T cells can transiently express FoxP3 under some circumstances [2]. FoxP3 maintains T_reg_ gene expression induced by other transcription factors rather than actually driving T_reg_ development. However, FoxP3 is essential for T_reg_ function since loss of FoxP3 function results in severe lymphoproliferative disease and autoimmunity in humans and mice [3]. The role of FoxP3 in maintaining self-tolerance was first identified in scurfy mice, and then in humans with immunodysregulation, polyendocrinopathy, enteropathy, X-linked (IPEX) syndrome, both of which have a FoxP3 mutation as the underlying genetic defect. Both natural and induced T_reg_cells (iT_regs_) have unique surface markers that differentiate them from conventional T cells; however differentiating nT_regs_ from iT_regs_ has been challenging. Additional subsets of iT_regs_ that have been described in humans and mice include Tr1 cells, T helper 3 cells (Th3), and CD8^+^ T_regs_ [4]. Tr1 and Th3 cells do not express FoxP3 but were considered “original” T_regs_ because of their suppressive function [2]. A number of studies have shown that T_regs _affect the magnitude of immunity and outcome of viral infections, especially with persistent viruses that give rise to chronic lesions [5]. The ability of viruses to induce proliferation and activation of regulatory T cells likely contributes to delayed clearance and persistence in the host.

Investigators have shown that both coronavirus and arterivirus infection results in an increase in CD4^+^CD25^+^FoxP3^+^ lymphocytes. However, the outcomes of T_reg_ activation for each virus are different. T_reg_ induction by porcine arterivirus suppresses the immune response [6] which may allow for viral persistence, while T_reg_ induction by neurotropic mouse hepatitis virus limits demyelination [7]. In this review we discuss the development of T_regs_ in the context of coronaviral and arteriviral infection with special emphasis on porcine arterivirus and mouse hepatitis virus.

## 2. Regulatory T Cell Development

### 2.1. Natural T_regs_

Natural regulatory T cells (nT_regs_) develop in the thymus through interactions between the high-affinity T cell receptor and cognate antigens on thymic epithelial cells. Co-stimulation through CD28 and common gamma (cγ) chain cytokines, especially IL-2 and IL-7 are necessary for nT_reg_ development. Signal transducer and activation of transcription 5 (STAT5) signaling through the cγ chain is also required. Thymic development of nT_reg_ cells follows a two-step process. First, thymocytes upregulate CD25 and other IL-2 signaling molecules in response to TCR/CD28 co-stimulation and second, CD4^+^CD25^high^FoxP3^-^ T_regs_ respond to IL-2 independent of the TCR, and induce FoxP3 expression in response to STAT5 activation [1]. T_reg_ cell-intrinsic NF-κB activation is essential for thymic T_reg_ development [1]. Naturally occurring T_regs_ cannot produce IL-2 and therefore rely on paracrine IL-2 production from conventional T cells. Mice deficient in IL-2 or CD25 have reduced numbers of FoxP3^+^ T cells and a dramatic reduction in peripheral and thymic T_regs_. In humans, Hassall’s corpuscles in the thymic medulla secrete thymic stroma lymphopoietin (TSLP) which activates immature dendritic cells and upregulates the expression of co-stimulatory molecules. The activated DCs induce FoxP3 expression in CD4^+^CD8^-^CD25^- ^thymocytes [8].

### 2.2. Inducible T_regs_

Less well-characterized are the inducible T_regs_ (iT_regs_) that develop from conventional T cells under certain conditions. iT_regs_ are induced by prolonged exposure to circulating antigen, chronic inflammation, or weak co-stimulation in the periphery [2]. Soluble factors such as the cytokines IL-4, IL-10 and TGF-β, retinoic acid or neuropeptides can upregulate FoxP3 expression and generate iT_regs_ in the periphery. Increased expression of FoxP3 results in upregulation of other T_reg_ molecules, including CTLA-4, GITR, and CD127. CD4^+^ T cells that express high levels of the IL-2α receptor (CD25^high^) do not respond to T cell receptor (TCR) activation or mitogen stimulation, and inhibit IL-2 transcription in CD25^-^ cells. Suppression of CD25^-^ cells is contact dependent, and requires activation of the T_regs _through the TCR; however, once activated, the suppressor effector function is nonspecific [9]. CD4^+^CD25^+^ T cells suppress the immune response to some viruses, protozoa, and bacteria, and aid the survival of intracellular pathogens [10] most likely by potent suppression of proliferation and IFN-γ production of both CD4^+^ and CD8^+^ T lymphocytes [11]. Tr1 cells secrete high amounts of IL-10 and moderate amounts of TGF-β. Inhibiting IL-10 with neutralizing antibody blocks the suppressor effects of Tr1 cells [4]. Th3 cells produce high concentrations of TGF-β and moderate amounts of IL-10, and the suppressor effects are not antigen specific [4]. Interestingly, Th3 cells suppress the activation of both Th1 and Th2 cell clones while other subsets primarily inhibit Th1 cells and have no effect on Th2 cells [4]. Other cells with adaptive regulatory function include some CD4^-^CD8^-^ and CD8^+^CD28^-^ T cells [8]. Early after iT_regs_ are stimulated, they express high levels of cell-cycle progression and T cell activation-associated genes [12], mimicking genes that are upregulated in activated effector T cells. As iT_regs_ mature, expression of these genes diminishes while they remain high in mature effector T cells. By 10 days after differentiation into iT_regs_, most cell cycle progression and T cell activation genes are expressed at levels approximately 3 times lower than in effector T cells. In addition, genes in the FoxO family of transcription factors are over-expressed in iT_regs_ compared to overexpression of the FoxM1 family in effector cells [12].

### 2.3. Viral Mediated-T_reg_ Activation

Regulatory T cells typically increase late in chronic viral disease to prevent a persistent inflammatory response and viral-mediated immunopathology. In fact, tissue-protective effects of T_reg_ were shown in models of respiratory syncytial virus, Friend virus, and West Nile Virus infection [13,14]. Additionally, T_reg_ responses to viruses (and bacteria) form the basis of the “hygiene hypothesis”. Infection with influenza A in suckling mice protected these mice as adults against allergy induced airway hyperactivity due to the expansion of allergen-specific regulatory T cells [15]. Conversely, T_regs_ are widely accepted as a key contributor in modulating the host immune response to viral infection. T_regs _are an important component in regulating the magnitude of the immune response to infection, thus preventing excessive inflammation and tissue damage. But, they can also be inappropriately induced by viruses in order to swing the balance of the immune response in favor of maintaining viral infection [16]. In this way T_regs_ contribute to persistent infection of many viruses including Friend virus, herpes simplex virus, hepatitis C virus, hepatitis B virus, human immunodeficiency virus, feline immunodeficiency virus, simian immunodeficiency virus, cytomegalovirus and Epstein-Barr virus [13,14,17,18,19,20,21,22,23,24,25,26].

## 3. Arteriviruses

### 3.1. Porcine Reproductive and Respiratory Syndrome Virus

Within a susceptible herd, reproductive failure due to PRRSV infection can range from sporadic abortions to abortion storms that may persist within the herd for up to 6 months [27]. Exposure late in gestation may manifest as late-term abortion, or stillborn, partially autolyzed, or mummified fetuses [27]. Neonatal infection results in severe dyspnea and tachypnea, and mortality of up to 100%, while disease in weaned pigs is primarily due to pneumonia and secondary bacterial or viral infections [27]. PRRSV infection is also associated with decreased local cellular immunity, resulting in increased susceptibility to secondary bacterial and viral infections [28,29,30]. Piglets infected with PRRSV in utero have a decreased innate immune response to bacterial pathogens [28]. In utero infection with PRRSV inhibits macrophage phagocytosis of *Salmonella* spp., and inhibits alveolar macrophage oxidative burst [28]. Additionally, both PRRSV infection and vaccination decreases the efficiency of vaccines against *Mycoplasma hyopneumoniae* [31] and porcine pestivirus [32]. Infection and vaccination with PRRSV induces a rapid, non-neutralizing antibody response, and an early, weak, non-specific gamma interferon (IFN-γ) response [33,34]. A PRRSV-specific T lymphocyte IFN-γ response does not appear until at least 2 weeks after infection, [35] gradually increases, and plateaus at 6 months postinfection. This IFN-γ is associated with a slow increase in neutralizing antibody [33,36]. Peak viremia and shedding occur before development of a protective neutralizing antibody and IFN-γ [36]. Acute infection is followed by persistence of lower levels of virus in lymphoid tissue and then clearance after several months [36]. The cause of the delayed IFN-γ and neutralizing antibody response resulting in persistent infection is unknown. The ability to induce a rapid IFN-γ response is important both for viral clearance and heterologous protection by vaccination [37,38]. Vaccine strains currently in use in the United States do not provide adequate heterologous protection, perhaps because of their inability to stimulate an adequate IFN-γ response. We hypothesized that one reason for the inadequate IFN-γ may be due to the ability of PRRSV to stimulate regulatory T cells*in vitro* [39].

PRRSV infection results in a significant upregulation of IL-10 expression in peripheral blood mononuclear cells and pulmonary alveolar macrophages *in vivo* and *in vitro* [40,41,42,43]. However, the ability of PRRSV to induce IL-10 appears to vary depending on strain and it was recently demonstrated that a highly virulent PRRSV strain did not induce IL-10 *in vitro* or *in vivo* [44,45,46]. The primary action of IL-10 is to inhibit inflammatory cytokines including IL-1β, IL-6, TNF, and IL-12, and antagonize the function of antigen presenting cells by decreased surface expression of MHC class I and II molecules and reduction of costimulatory molecules [47]. Additionally, IL-10 inhibits the production of nitric oxide by macrophages [48,49]. Since IL-12-mediated production of IFN-γ by pulmonary alveolar macrophages reduces PRRS viral titers in the lungs and serum [50], inhibition of IL-12 synthesis by IL-10 likely enhances the occurrence of natural disease. IL-10 also affects innate immunity by inhibiting the response to Toll-like receptors (TLR), including TLR7 and TLR8 that recognize single-stranded viral RNA [51]. IL-10 treated DCs induce the proliferation of regulatory T cells (T_regs_) as well as antigen-specific anergy in CD4^+^ and CD8^+^ T lymphocytes [47]. IL-10 not only plays a role in the development of type 1 T_regs_ (Tr1), but is also one of the primary mechanisms by which T_regs_ inhibit effector T lymphocyte function [51].

In previous experiments, we have shown that PRRSV infection increases the number of CD4^+^CD25^+^FoxP3^+^ regulatory T cells in the lungs and PBMCs of pigs (LeRoith, unpublished). Silva-Campa demonstrated that the CD25^+^FoxP3^+^ cells induced by PRRSV produce TGF-β, suggesting that the cells are not only increased, but are also functional [39]. These CD25^+^FoxP3^+^ T_regs_ exhibited suppressor activity *in vitro*. This same group reported that while American genotype PRRSV strains were capable of inducing T_reg_ and upregulating TGF-β production, DCs infected with European genotype PRRSV induced neither TGF-β nor T_regs_ [52]. Wongyanin and colleagues demonstrated that peripheral blood mononuclear cells (PBMCs) cultured with American genotype PRRSV *in vitro* induced virus-specific CD4^+^CD25^+^FoxP3^+^ T_regs_ and the addition of monocyte-derived dendritic cells (MoDC) to the cell culture enhanced T_reg_ induction [53]. Not only was there a significant increase in the numbers of CD4^+^CD25^+^FoxP3^+^ cells, but these PRRSV-specific T_regs_ exhibited suppressive activity when co-cultured with PHA-stimulated autologous peripheral leukocytes. The authors reported in this study that PBMCs collected from PRRSV-infected pigs 10 days post-inoculation exhibited significantly higher numbers of CD4^+^CD25^+^FoxP3^+^ lymphocytes when cultured in the presence of PRRSV compared to mock-infected cell lysate or PBMCs alone. The role of PRRSV nucleocapsid protein (N) in T_reg_ induction was recently described, and it was found that N-protein induced IL-10 producing cells and CD4^+^CD25^+^FoxP3^+^ T_regs_ in a DC *in vitro* system [54]. In this study, T_reg_ induction was found to be dependent, at least in part, on IL-10, as neutralization of IL-10 by anti-IL-10 antibody drastically reduced the PRRSV-induced T_regs_.

In order to assess the potential benefit of using a mucosal rather than parenteral immunization approach, Dwivedi *et al.* investigated the ability of an intranasally delivered PRRS-modified live vaccine (MLV) augmented with a potent *Mycobacterium tuberculosis* whole cell lysate adjuvant to induce cross protective immunity against a heterologous PRRSV strain. Vaccinated pigs had a reduced frequency of T_regs_ in respiratory mucosal and systemic sites compared to unvaccinated pigs, and this correlated with decreased secretion of immunosuppressive cytokines IL-10 and TGF-β, diminished lung pathology, and increased PRRSV neutralizing antibody titers and IFN-γ secretion [55,56]. These findings suggest that the route of immunization and adjuvant-mediated immunomodulation may influence T_reg_ dynamics, thereby facilitating or negating efficient viral clearance. This same group recently reported on pigs that that were maintained on a commercial farm and experimentally infected with PRRSV.

At two days post-inoculation, infected pigs had an increased frequency of circulating CD4^+^CD25^+^Foxp3^+^ T_regs_, reduced frequency of CD4^-^CD8^+^ and CD4^+^CD8^+^ T cells, and enhanced IL-10, IL-4 and IL-12 secretion [57].

It is unknown which components of the virus are responsible for T_reg_ proliferation, but this is currently under investigation by our group. We demonstrated that T_reg_ induction by PRRSV results in increased susceptibility to natural *Mycoplasma hyopneumoniae* infection [6]. In this study, pigs were inoculated with a virulent strain of PRRSV and a modified live vaccine derived from the same strain. The attenuated strain contains silent mutations in the replicase region (ORF 1) [58] and conservative or non-conservative amino acid changes in the structural proteins [58]. We hypothesized that even with changes that decrease pathogenicity, the mutations would not alter the virus’s ability to stimulate T_regs_. Consistent with our hypothesis, we found that, although attenuated, the vaccine strain did not differ from the parent strain in its ability to activate T_regs_. The animals inoculated with the attenuated vaccine did not differ from animals inoculated with the parent strain in the severity of *M. hyopneumonia*-mediated disease [6]. Similar to previous findings that infection with wild type PRRSV or vaccination with PRRS MLV vaccines has been shown to decrease the efficacy of *M. hyopneumoniae* vaccines [31], inoculation with each of the three PRRS viruses in this study resulted in activation of regulatory T cells and likely decreased the ability of the pigs to mount an effective anti-bacterial immune response.

Vaccine efficacy appears to be related to the ability to stimulate IFN-γ production and efficacy against heterologous virus challenge seems to correlate more with the ability to stimulate IFN-γ than homology of the vaccine strain to the infective strain. Current vaccines fail to protect against other strains [59], which may be due to their inability to stimulate IFN-γ production. Our study was the first to show the correlation between vaccine induction of T_regs_ and increased susceptibility to bacterial infection. Although T_regs_ that are induced by PRRS *in vitro* produce TGF-β [39], the induction of T_regs_ may indirectly result in IL-10 production, a phenomenon that is well established in the PRRS literature [41,42]. Production of IL-10 instead of IFN-γ by the MLV vaccine strain would lead to a lack of heterologous protection, and decreased efficacy of other vaccines, as seen by other authors [31]. Our results suggest that mutations in the vaccine strain that result in attenuation of the virus do not alter the virus’s ability to stimulate T_regs_ [6]. This information can help us design vaccines in which the T_reg_-stimulating epitopes are mutated or deleted in order to stimulate a robust virus-specific IFN-γ response, and provide protection against heterologous strains [6].

### 3.2. Lactate Dehydrogenase Elevating Virus

Lactate dehydrogenase-elevating virus (LDV) has been described as an “ideal” persistent virus since it is associated with life-long viral infection in mice in the absence of clinical disease while escaping the host immune response [60,61]. Cytotoxicity is limited to resident tissue macrophages, in which viral replication is maintained. Lifelong immunotolerance is maintained and has been shown to be due in part to continuous generation of LDV antigens in the thymus [62]. In addition, LDV infection and replication within macrophages appears to be robustly resistant to typical antiviral immune responses including interferon-α/β, antiviral antibodies and virus-specific cytotoxic T lymphocytes [60]. Collectively, these features suggest a role for T_regs_ in the pathogenesis of LDV infection.

Inada and colleagues demonstrated that mice challenged with heat-inactivated or killed LDV induced a strong virus-specific delayed-type hypersensitivity (DTH) reaction, whereas a delayed hypersensitivity reaction was undetectable in young (1–3 month-old) mice challenged with live virus [63]. In these experiments pretreatment with cyclophosphamide partially restored the DTH response, which was attributed to cyclophosphamide mediated elimination of suppressor T cells [64,65]. Live virus also elicited a DTH response in old mice (> 8 months) without cyclophosphamide treatment, which is not surprising in light of the fact that older mice have reduced suppressor T cell activity. The cumulative findings from this study suggest that inoculation with live LDV induces suppressor T cells, and that this dampens the virus-specific delayed hypersensitivity response [63].

Further demonstrating the immunosuppressive ability of LDV, Robertson, *et al.* reported that co-infection with LDV and Friend virus (FV) delayed the FV specific CD8^+^ T cell response and that this resulted in an increase in duration and severity of the acute phase of FV infection [66]. The suppressed FV-specific CD8^+^ T cell response occurred in mice acutely co-infected with LDV and FV as well as in mice inoculated with LDV 8 weeks prior to FV infection. In addition, mice infected with LDV exhibited significant regulatory T cell-mediated suppression of IFN-γ production by FV-specific CD8^+^ T cells that peaked at day 3 post-infection and was diminished by day 7 post-infection. However, failure of FV/LDV co-infected mice to mount a strong CD8^+^ T cell response was not attributed solely to T_reg_-mediated suppression, because neither depletion of CD4^+^ cells nor pre-treatment with anti-CD25 antibody restored the normal CD8^+^ response [66].

The antigen presenting ability of spleen, lymph node and peritoneal macrophages from LDV infected mice was shown to be diminished as measured *in vitro* by reactivation of memory T cells. The experiments showed that LDV-mediated impairment of antigen presentation was not due to diminished uptake of antigen by macrophages and LDV-infected peritoneal macrophages were not immunosuppressive in cell culture. Rather, the authors concluded that the decrease in antigen presenting ability of LDV-infected macrophages was related to reduced expression of Ia antigen or virus-mediated elimination of Ia positive macrophages from the peritoneum [67].

### 3.3. Coronavirus

Much attention on the interaction of T_regs_ and viral pathogens has focused on chronic disease, demonstrating the ability of T_regs_ to delay or prevent viral clearance leading to persistent infection [16,68]. From the host point-of-view, virus-induced T_regs_ represent a detrimental factor in the context of chronic or persistent infection. However, coronavirus infection in the central nervous system of mice highlights a beneficial role of T_regs_ in reducing bystander damage as a consequence of acute infection. Coronavirus infection of the central nervous system exemplifies the necessity of a delicately balanced and finely orchestrated immune response. While a rapid and strong pro-inflammatory response aids in viral clearance, host tissue destruction secondary to immunopathology can be a deleterious side effect. An appropriate anti-inflammatory response is necessary for minimizing collateral damage while still allowing clearance of the invading pathogen.

Mice infected with the neurovirulent strain of mouse hepatitis virus JHM (JHMV) develop a rapidly progressive, fatal disease that has been shown to be mediated, in part, by CD4^+^ T cells. This was evidenced by the fact that infection with a recombinant JHMV strain containing a single mutation in an immunodominant CD4^+^ T cell epitope (rJ.M_Y135Q_) resulted in nonlethal mild encephalitis, and the decrease in mortality correlated with decreased numbers of virus-specific CD4^+^ T cells in the brain [69]. Further investigations found that significantly higher levels of the pro-inflammatory cytokines/chemokines IL-6, CCL2, CCL5 and IFN-γ were detected in the brains of mice infected with the non-mutated recombinant JHMV strain (rJ) compared to rJ.M_Y135Q_-infected mice [70]. CD4^+^ T_regs_ were shown to be critical in ameliorating disease because (1) greater numbers of T_regs_ were detected in the brains of rJ.M_Y135Q_-infected mice compared to rJ-infected mice, (2) their depletion in rJ.M_Y135Q_-infected mice increased morbidity and mortality, and (3) adoptive transfer of T_regs_ into rJ-infected mice increased survival from 0% to 50% [70]. The authors concluded that in the setting of acute encephalitis, T_regs_ may aid in limiting immunopathology, thus decreasing clinical disease without delaying viral clearance.

In contrast to the neurovirulent JHMV strain, mice infected with an attenuated JHMV variant (J2.2-V-1) develop chronic demyelinating encephalomyelitis and the demyelination is largely due to immunopathology associated with viral clearance [71,72,73]. Trandem *et al.* demonstrated that adoptive transfer of T_regs_ ameliorated clinical disease and demyelination in J2.2-V-1-infected mice and did not delay viral clearance in immunocompetent C57BL/6 mice [74]. The authors provided evidence that this improved clinical outcome was due in part to T_regs_ functioning in the draining cervical lymph nodes to suppress T cell proliferation, dendritic cell activation and expression of pro-inflammatory mediators. These findings further support the notion that T_regs_ play an important role in limiting neuropathology associated with mouse hepatitis virus (MHV) infection while still allowing for viral clearance.

Identification and characterization of pathogen-specific epitopes targeted by T_regs_ has received considerable interest due to possible therapeutic interventions aimed at diminishing inflammation-induced tissue damage in a pathogen-specific fashion. Zhao and colleagues identified T_regs_ that specifically recognize two mouse hepatitis virus-specific epitopes using the neurotropic rJ2.2 strain of mouse hepatitis virus, which is known to cause mild acute encephalitis and chronic demyelination [7,75]. These virus-specific T_regs_ were present in the virus-infected central nervous system and, based on concurrent detection with virus-specific effector CD4^+^ T cells and identification within the naïve T cell precursor pools in the spleen and lymph nodes, are presumed to arise from the natural T_reg_ pool. In addition, the virus-specific T_regs_ expressed IL-10 and IFN-γ upon stimulation with viral peptide and suppressed proliferation of cognate-epitope specific effector CD4^+^ T cells. While T_regs_ are known to play a critical role in reducing immunopathology and clinical disease in rJ2.2-infected mice, the results from this study suggest that virus-specific T_regs_ may be significantly more potent in diminishing immunopathology associated with encephalomyelitis compared to adoptive transfer of natural T_regs_, particularly during acute infection when maximum viral antigen is present [7,74,76].

In contrast to the protective effect of T_regs_ in the pathogenesis of neurotropic mouse hepatitis virus infection, Shalev and colleagues demonstrated that T_regs_ contributed to more severe fulminant viral hepatitis in susceptible mice infected with murine hepatitis virus strain 3. This phenomenon was mediated in part by increased T_reg_ expression of the immunosuppressive cytokine fibrinogen-like protein 2 [77]. These findings suggest that T_reg_ activation following infection with mouse hepatitis virus may culminate in different outcomes depending on the anatomic location of disease, such that they are paradoxically beneficial to the host in limiting CNS disease but harmful in potentiating fulminant hepatitis.

## 4. Conclusions

Regulatory T cells are critical for maintaining immune tolerance and immune homeostasis by protecting against devastating autoimmune disease and overwhelming inflammation. Without these subsets of T cells, animals quickly succumb to inflammatory or autoimmune diseases. Activation of T_regs_ is critical in some viral infections, especially neurotropic coronaviral infection, to limit immunopathology. However, some viruses, including arteriviruses, have exploited these cells to enhance their replication in the host and become persistent. The function of T_regs_ in coronavirus-infected mice highlights the potential dichotomy in protective versus harmful outcome, depending on the anatomic location of disease. Along this line of reasoning, one might argue that the perspective of T_regs_ protecting the host or enhancing disease may depend on which outcome is more deleterious, viral persistence or immunopathology associated with viral clearance.

Understanding the mechanism of T_reg_ activation by viruses is critical for identifying new strategies to prevent the immunosuppressive effects or enhance the immunoprotective effects on the host. In many cases, activation of T_regs_ not only dampens the immune response to the virus, but non-specifically dampens the immune response to other pathogens. While the initial immune suppression likely plays a role in virus persistence, the non-specific immune suppression is one of the mechanisms by which secondary infection can occur. The complete effects of these viruses on the immune response of the host are still under investigation. The ability of certain viruses to stimulate T_regs_ provides valuable insight as to how viruses modulate the immune system. Understanding the mechanisms of T_reg_ induction is important in determining the contribution of these viruses to the development of disease and is also essential for vaccine development. 
